# General Medical Council and the Supply of Doctors

**Published:** 1915-06-19

**Authors:** 


### GENERAL MEDICAL COUNCIL AND THE SUPPLY OF DOCTORS.

The General Medical Council has large public responsibilities, and the present national emergency has rendered some of these particularly acute. Though the Council has no direct power to modify the numbers entering the medical profession it cannot fail to be concerned when from any cause there is a risk that the supply of doctors may prove unequal to the general welfare. From various causes, but more particularly from the exceptional demands made by the Army, this risk has now become a reality. It is therefore no matter for surprise that at the recent session of the Council prominence was given to means by which the existing failure can be met. That it can be fully and satisfactorily met is evidently impossible. When we know that the number of medical students at the end of 1914 was less by more than a thousand than in the previous year a serious deficiency in the immediate future is inevitable, while the urgency of the moment is notorious. The steps taken by the Council to relieve the situation are helpful, though they only reach the fringe of the position. In the first place the recall of senior students who had volunteered for active service has secured an increase of the numbers who will be able within the next few months to obtain diplomas as qualified medical practitioners, and this is an important and substantial gain. Secondly, under the powers conferred upon them by the Medical Act of 1886, and with the approval of the Belgian Government and the Privy Council, the Medical Council has officially recognised the diplomas of certain of the Belgian Universities, with the result that some sixty of the holders of these diplomas are now legally qualified to practise in the United Kingdom and to undertake professional work in hospitals and other institutions. A third proposal, though the formalities essential to this are not quite complete, is the removal of certain restrictions which at present hinder full medical reciprocity between this country and certain of the Provinces of the Dominion of Canada. The project is being actively forwarded, and ere long we may confidently expect that all Canadian graduates will be recognised as qualified to take service on behalf of the State and in any part of His Majesty's Dominions, as are already the medical graduates of the several universities of the Commonwealth of Australia. Apart from its mere medical value, such an arrangement in the present circumstances has a special interest as a practical application of Imperial federation in an important field of activity.

To the emergency of the moment the Council can make but little contribution. The production of doctors is a long and costly process, and it cannot be hastened by stamping on the ground. Quality is not less important than quantity, and one of the public duties of the Council is to see that the quality is maintained. Hence it is not inopportune, in view of the pressure of the day, that the medical corporations and schools should be reminded of the necessity of maintaining the existing standards both of teaching and of examination. Weakness in either of these respects would only mean future disaster, and no emergency would justify it. The one definite proposal made in this direction has been the suggestion that qualified pharmacists should be relieved of certain examination tests. We dealt with this matter in our issue of May 15 (p. 158), and we are gratified to find that our views have been endorsed by the Medical Council. That the medical and pharmaceutical courses of study coincide in a certain limited sphere is quite true, but the point of view is not the same in the two cases, and the contemplation of the one cannot be an effective substitute for the regulated study of the other.

